# Mapping global evidence on public-private partnership for medical rehabilitation services delivery: a scoping review protocol

**DOI:** 10.1186/s13643-022-02155-4

**Published:** 2023-01-04

**Authors:** Senzelwe Mazibuko, Thayananthee Nadasan, Pragashnie Govender

**Affiliations:** Department of Physiotherapy, School of Health Sciences, College of Health Sciences, Westville Campus, University of KwaZulu-Natal, 10 Maxwell Street, Empangeni, South Africa

**Keywords:** Public-private partnership, Medical rehabilitation, Physiotherapy, Scoping review

## Abstract

**Introduction:**

Access to medical rehabilitation remains poor in Sub-Saharan Africa. This is partly due to inadequate service delivery emanating from ill-defined public health policies. Developed countries have adopted public-private partnership (PPP) agreements between the government and private sectors, thus presenting superior quality and access to rehabilitation services. To help develop a PPP model for physiotherapy service delivery in South Africa, this scoping review will map research linked to PPP for medical rehabilitation services delivery and outcomes in the global context.

**Methodology:**

The Arksey and O’Malley (2005) framework (identify the research question, identify relevant research, select studies, chart the data, collate, summarize, and report findings) will be used to guide this review. Peer-reviewed literature will be searched in PubMed, EBSCOhost, Cochrane library, SCOPUS, and Google Scholar from 2000-2022 using a combination of keywords, Medical Subject Headings, and Boolean terms. Screening of the articles at all stages will be conducted independently by two reviewers using the eligibility criteria as a guide. The reference lists of retrieved articles will be manually searched for relevant studies. Emerging themes and sub-themes will be collated, summarized, and the results reported in the narrative form.

**Discussion:**

We anticipate identifying literature gaps for future research to inform policy on PPP for rehabilitation services delivery in Sub-Saharan Africa and actual practice. The results of this review will contribute to building a model that will enable the provision of equitable rehabilitation services at the district health level using PPP.

## Background

The World Health Organization’s (WHO) *59-Nation Report on Disability* shows that 15.6% of the adult population live with some form of physical disability. Of these, 19.2% are female, and 12.0% are male. The prevalence of disability in high-income countries is 11.8%. In comparison, that of low-income countries is 18% [1]. In low-income countries; women make up the majority (22.1%) of people living with disabilities (PLDs) while males make up 13.8% [1]. On the wealth quintile scale, where Q1 (poorest) and Q5 (richest), 20.7% of PLDs come from the poorest quintile while Q5 accounts for 11.0% of PLDs [1]. In low-income nations, PLDs make up 22.4% of Q1, while 13% of PLDs come from Q5 [1]. In high-income countries, this disparity is more pronounced with 17.6% of PLDs being in Q1 and only 6.5% of PLDs being within Q5 [1]. Thus, a majority (16.4%) of PLDs reside in rural areas while 14.6% reside in urban areas, suggesting that disability is more prevalent in rural settings with a higher rate of poverty compared to urban areas [1]. Previous studies have shown the prevalence of disability and poverty have a reciprocal relationship, which is exacerbated by the poor or no healthcare services in rural areas [2–5].

The United Nations Convention on the Rights of Persons with Disabilities (UNCRPD) and other policy such as the National Rehabilitation Programme (NRP) stipulate that PLDs have a right to quality healthcare. However, health services to PLDs have been hampered by lack of human resources for health, dilapidated infrastructure, overburdened public health systems, and finite financial resources [5–7]. In sub-Saharan Africa (SSA), PLDs have limited access to rehabilitation services [8]. This has been attributed to a lack of funding to build rehabilitation centers and high financial costs associated with rehabilitation [8]. Furthermore, referral pathways are irregular, and the availability of rehabilitation services are compromised, and thus, patients suffer avoidable complications because of inadequate follow-up [5, 7–10]. Lack of infrastructure compounds the rehabilitation challenges, particularly in SSA, where district hospital rehabilitation units are poorly maintained and scarce [9]. There is little to no informative research on appropriate rehabilitation development indicators at the tertiary, specialized, or primary healthcare (PHC) level [9]. Currently, poorly resourced regions depend on community-based rehabilitation (CBR), friends, family, and other community groups [1].

The PPP model has been identified as a key strategy to enhance public health systems and mitigate the rising costs of an already expensive and unsustainable private healthcare sector [11, 12]. A PPP is an agreement between a government institution and a private party, where (i) the private party performs an institutional function and/or uses state property in terms of output specifications and (ii) substantial project risk (financial, technical, operational) is transferred to the private party, and the private party benefits through unitary payments from government budgets and/or user fees [13].

There has been an increasing interest to implement PPPs among other Sub-Saharan countries in the effort to improve public health systems [14–18]. In Lesotho, the private sector was involved in the refurbishment and redevelopment of Queen Mamohato Memorial Hospital in Maseru, a public institution [19]. In Uganda, the government partnered with a local church to construct Ruharo Mission Hospital [20]. Therefore, it is evident that PPPs may leverage resources by collaborating in mutually beneficial partnerships with private healthcare providers to create an effective, efficient, and responsive public health sector through the transfer of private-sector technical skills, innovation, and resources [12, 21, 22]. Government is tasked with improving their health systems to meet the needs of majority of citizens who depend on public health. To help develop a PPP model for physiotherapy service delivery in South Africa, this current scoping review will map research linked to PPP for medical rehabilitation services delivery and outcomes in the global context.

## Methodology

Preferred Reporting Items for Systematic Reviews and Meta-Analyses guideline for Protocols (PRISMA-P) was followed to develop this protocol [23]. This review study will use the following steps: identifying the research question; identifying relevant studies; study selection; charting the data; and collating, summarizing, and reporting results outlined by Arksey and O’Malley’s [24] in their methodological framework.

### Identifying research question(s)

The primary research question for this review is “What research evidence linked to PPP and medical rehabilitation service delivery in SSA exist?” The population, concept, and context for this review question are defined in Table 1. The secondary research question for this review will be “What PPP models PPP for medical rehabilitation services delivery exist globally?”.

**Table 1:** Population, concept and context framework for the main review question

Population	Individuals of all ages using medical rehabilitation services such as occupational therapy, physiotherapy, speech and audiology
Concept	Public-private partnership: This refers to a contract between a private party and a government agency for providing a public service, in which the private party bears significant risk and management responsibility [25]
Context	Sub-Saharan Africa: This will include countries in the WHO Africa Region

### Identifying relevant studies

Relevant peer-reviewed articles and unpublished literature (grey literature) in the English language published in the last twenty years (from 2000 to 2022) will be sourced from electronic databases. The databases will include PubMed, EBSCOhost (Academic search complete, CINAHL with full text, Health Sources), Cochrane Library, SCOPUS, and Google Scholar. In consultation with an expert librarian, a search strategy will be developed using keywords, Boolean terms (AND/OR), and Medical Subject Heading terms. The keywords will include the following: “medical rehabilitation,” “physical therapy,” “physiotherapy,” “occupational therapy,” “speech therapy,” “public-private partnership,” “public-private mix,” “public-private cooperation,” “public-private coordination,” “public-private collaboration,” “contract out,” “contracting out,” “Private finance initiative contracts.” We will adequately document each search strategy as illustrated in Table 2 (pilot search strategy in PubMed). The principal author (SMM), a physiotherapist, will conduct the database search assisted by the rest of the review team and import all articles to an EndNote library created for the study. We will additionally search the reference list of the included articles for relevant evidence sources.

**Table 2:** A pilot search strategy conducted in PubMed database

Date	Database	Keywords	Search results
10/09/2022	PubMed	(((((“rehabilitant”[All Fields] OR “rehabilitants”[All Fields] OR “rehabilitate”[All Fields] OR “rehabilitated”[All Fields] OR “rehabilitates”[All Fields] OR “rehabilitating”[All Fields] OR “rehabilitation”[MeSH Terms] OR “rehabilitation”[All Fields] OR “rehabilitations”[All Fields] OR “rehabilitative”[All Fields] OR “rehabilitation”[MeSH Subheading] OR “rehabilitation s”[All Fields] OR “rehabilitational”[All Fields] OR “rehabilitator”[All Fields] OR “rehabilitators”[All Fields])) OR (“physical therapy modalities”[MeSH Terms] OR (“physical”[All Fields] AND “therapy”[All Fields] AND “modalities”[All Fields]) OR “physical therapy modalities”[All Fields] OR “physiotherapies”[All Fields] OR “physiotherapy”[All Fields]) OR (“occupational therapy”[MeSH Terms] OR (“occupational”[All Fields] AND “therapy”[All Fields]) OR “occupational therapy”[All Fields]) OR (“speech therapy”[MeSH Terms] OR (“speech”[All Fields] AND “therapy”[All Fields]) OR “speech therapy”[All Fields])) AND (“public private sector partnerships”[MeSH Terms] OR (“public-private”[All Fields] AND “sector”[All Fields] AND “partnerships”[All Fields]) OR “public private sector partnerships”[All Fields] OR (“public”[All Fields] AND “private”[All Fields] AND “partnership”[All Fields]) OR “public private partnership”[All Fields])) OR (“public-private”[All Fields] AND “mix”[All Fields]) OR (“public private sector partnerships”[MeSH Terms] OR (“public-private”[All Fields] AND “sector”[All Fields] AND “partnerships”[All Fields]) OR “public private sector partnerships”[All Fields] OR (“public”[All Fields] AND “private”[All Fields] AND “cooperation”[All Fields]) OR “public private cooperation”[All Fields]) OR (“public-private”[All Fields] AND (“coordinate”[All Fields] OR “coordinated”[All Fields] OR “coordinately”[All Fields] OR “coordinates”[All Fields] OR “coordinating”[All Fields] OR “coordination”[All Fields] OR “coordinations”[All Fields] OR “coordinative”[All Fields] OR “coordinatively”[All Fields] OR “coordinator”[All Fields] OR “coordinator s”[All Fields] OR “coordinators”[All Fields])) OR (“public-private”[All Fields] AND (“collaborate”[All Fields] OR “collaborated”[All Fields] OR “collaborates”[All Fields] OR “collaborating”[All Fields] OR “collaboration”[All Fields] OR “collaborations”[All Fields] OR “collaborative”[All Fields] OR “collaborative s”[All Fields] OR “collaboratively”[All Fields] OR “collaboratives”[All Fields] OR “collaborator”[All Fields] OR “collaborators”[All Fields])) OR ((“contract s”[All Fields] OR “contracted”[All Fields] OR “contractibility”[All Fields] OR “contraction”[All Fields] OR “contractional”[All Fields] OR “contractions”[All Fields] OR “contractive”[All Fields] OR “contractivity”[All Fields] OR “contracts”[MeSH Terms] OR “contracts”[All Fields] OR “contract”[All Fields] OR “contracting”[All Fields]) AND “out”[All Fields]) OR ((“contract s”[All Fields] OR “contracted”[All Fields] OR “contractibility”[All Fields] OR “contraction”[All Fields] OR “contractional”[All Fields] OR “contractions”[All Fields] OR “contractive”[All Fields] OR “contractivity”[All Fields] OR “contracts”[MeSH Terms] OR “contracts”[All Fields] OR “contract”[All Fields] OR “contracting”[All Fields]) AND “out”[All Fields]) OR ”private"[All Fields] OR ”privately”[All Fields] OR “privates”[All Fields] OR “privatization”[MeSH Terms] OR “privatization”[All Fields] OR “privatizations”[All Fields] OR “privatize”[All Fields] OR “privatized”[All Fields] OR “privatizing”[All Fields]) AND (“economics”[MeSH Subheading] OR “economics”[All Fields] OR “finances”[All Fields] OR “economics”[MeSH Terms] OR “financing”[All Fields] OR “finance”[All Fields] OR “financed”[All Fields] OR “financer”[All Fields] OR “financers”[All Fields] OR “financings”[All Fields]) AND (“initiative”[All Fields] OR “initiative s”[All Fields] OR “initiatives”[All Fields]) AND (“contract s”[All Fields] OR “contracted”[All Fields] OR “contractibility”[All Fields] OR “contraction”[All Fields] OR “contractional”[All Fields] OR “contractions”[All Fields] OR “contractive”[All Fields] OR “contractivity”[All Fields] OR “contracts”[MeSH Terms] OR “contracts”[All Fields] OR “contract”[All Fields] OR “contracting”[All Fields]))) AND (y_10[Filter])	4210

### Eligibility criteria

This scoping review has a criterion to select most relevant studies to answer the research question(s). Thus, the inclusion and exclusion criteria are listed below.

#### Inclusion criteria

- All articles that focus on medical rehabilitation services (occupational, physiotherapy, speech and audiology, psychologists, social workers, and dieticians/nutritionist),
- Articles that include PPP for medical rehabilitation service,
- Articles presenting evidence on access to medical rehabilitation services,
- Articles presenting evidence on referral pathways in medical rehabilitation
- Articles showing PPP models/frameworks for medical rehabilitation services,
- Primary study designs and frameworks/models,
- English language, and
- Publication from 2000 to 2022.

#### Exclusion criteria

- Articles that exclude medical rehabilitation practitioners,
- Articles focusing on non-medical rehabilitation, such as drug rehabilitation.
- Studies that focus on access to other healthcare services,
- Scoping review, systematic reviews, and meta-analysis, literature reviews without PPP model/framework for medical rehabilitation, and
- Articles published before 2000.

### Study selection

**Fig. 1:**
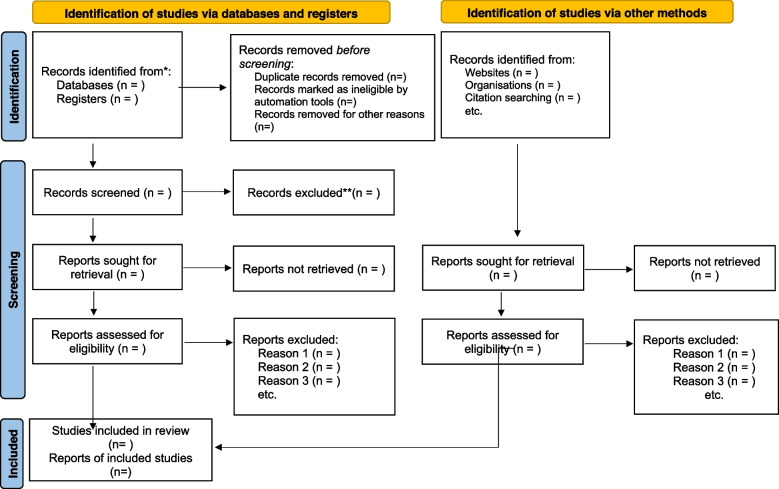
PRISMA 2020 flow diagram

The EndNote library will be cleaned by identifying and removing all duplicate articles and shared with the review team. The screening tools will be piloted and tested by two reviewers independently and the necessary adjustments based on the feedback received to ensure the screening tools are accurate and reliable. Two reviewers will screen the titles and abstracts and the full-text articles independently. Based on the eligibility criteria, the two independent reviewers will sort the articles into either the “include” or “exclude” group. Any differences in the responses of the two independent reviewers at the abstract screening stage will be resolved through discussion among the review team, but a third reviewer will be engaged to resolve any discrepancies at the full-text screening phase. The University of KwaZulu-Natal library services to retrieve full-text articles that are closed access publications, but emails will also be sent to the original authors or corresponding author to request relevant full-text articles if needed. PRISMA flow diagram will be used to account for the articles (Fig. 1).

### Charting data

A data extraction form will be developed for the charting of relevant data from the included articles (Table 3). Two reviewers will extract the data from the included studies independently using a pilot-tested form and a third reviewer employed to resolve any discrepancies that arise. We will extract the bibliographic characteristics of the included studies (author(s), year of publication, country, study title, study aim/objective, study population), gender of study participants, study setting (hospital or clinic or community-based). Moreover, we will extract results relating to PPP and medical rehabilitation services such frameworks/models, referral pathway, availability, human resource capacity, perceptions, and experiences of PPP-based intermediate medical rehabilitation. In addition, we will extract all other data from the conclusion and/or recommendations of the included articles to answer this study’s question.

**Table 3:** Proposed data extraction form

Author and publication year	
Study/article title	
Study objective	
Study design	
Country	
Study population	
Type of medical rehabilitation service	
Type of PPP model	
Significant findings	
Other relevant findings	
Conclusions and recommendations	

### Collating, summarizing, and reporting the results

A narrative synthesis will be used to summarize all relevant data into themes and sub-themes to answer this study question. The findings will be categorized into four themes (models exist for PPP medical rehabilitation services delivery, referral pathway for medical rehabilitation services, availability of medical rehabilitation service, human resource capacity for medical rehabilitation service, affordability of medical rehabilitation services, experiences and perceptions of PPP-based intermediate medical rehabilitation) and reported. Other relevant emerging themes or sub-themes will be reported. Tables and figures/maps will also be used to present the characteristics of the included studies, study results, and study findings that were appropriate.

## Discussion

This scoping review will map existing models for PPP for medical rehabilitation in SSA and the availability, affordability, and human resource capacity for rehabilitation in SSA. Equitable access to quality medical rehabilitation requires improvement [26]. A growing number of developing economies implement the PPP method to tackle critical infrastructural development services. The technical and innovative knowledge from the private sector is leveraged for said private partner to shoulder risk and create infrastructure that will ultimately be government property. Health systems in developing African nations require strengthening, and partnerships between government and the private sector can contribute to this effort [25, 26]. This work anticipates that rehabilitation will be shown to be the practical tool for providing healthcare through the district health system (DHS) [27]. Rehabilitation will be shown to be compatible with the ideology of holistic and preventative health. The literature may reveal gaps between the current rehabilitation policy of African countries and the current practices, due to the high economic inequality. This review is part of an ongoing study that intends to create a model for providing equitable, quality, and timeous physiotherapy services to the most-needed through the DHS, using PPP-based techniques. To this end, it will be relevant to draw evidence from related professional topic areas or fields, hence the reason for this study’s limitation to rehabilitation services only. This review will contribute to improving the quality assurance of the health system of South Africa and contribute to the research that will inform further relevant research in the region.

## Data Availability

We have duly cited all studies and data is presented in a form of references.

## Abbreviations

NRP: National Rehabilitation Policy
PHC: Primary healthcare
PLD(s): Persons living with disability/disabilities
PPP: Public-private partnership(s)
UNCRPD: United Nations Convention on the Rights of Persons with Disabilities
SSA: Sub-Sahara Africa
WHO: World Health Organization
DHS: District health system
CBR: Community-based rehabilitation

## References

[1] World Health Organisation. World Report on Disability. Geneva: World Health Organisation; 2011. Available from: https://www.who.int/disabilities/world_report/2011/report.pdf. Cited 2021 17/04/2021.

[2] Dayal H (2010). Provision of rehabilitation services within the District Health System-the experience of rehabilitation managers in facilitating this right for people with disabilities. S Afr J Occup Ther.

[3] Mji G, Chappell P, Statham S, Mlenzana N, Goliath C, De Wet C (2013). Understanding the current discourse of rehabilitation: With reference to disability models and rehabilitation policies for evaluation research in the South African Setting.

[4] M'Kumbuzi VRP, Myezwa H (2016). Conceptualisation of community-based rehabilitation in Southern Africa: a systematic review. S Afr J Physiother.

[5] Sherry K (2014). Disability and rehabilitation: essential considerations for equitable, accessible and poverty-reducing health care in South Africa. S Afr Health Rev.

[6] Hanass-Hancock J, Nene S, Deghaye N, Pillay S (2017). ‘These are not luxuries, it is essential for access to life’: disability related out-of-pocket costs as a driver of economic vulnerability in South Africa. Afri J Disabil.

[7] Visagie S, Swartz L (2016). Rural South Africans’ rehabilitation experiences: case studies from the Northern Cape Province. S Afr J Physiother.

[8] Naidoo U, Ennion L (2019). Barriers and facilitators to utilisation of rehabilitation services amongst persons with lower-limb amputations in a rural community in South Africa. Prosthetics Orthot Int.

[9] Health NDo (2015). Framework and strategy for disability and rehabilitation services in South Africa 2015–2020.

[10] Visagie S, Scheffler E, Schneider M (2013). Policy implementation in wheelchair service delivery in a rural South African setting. Afr J Disabil.

[11] Myezwa H, Van Niekerk M (2013). National Health Insurance implications for rehabilitation professionals and service delivery. S Afr J Physiother.

[12] Raman AV, Björkman JW (2015). Public-private partnerships in healthcare. the palgrave international handbook of healthcare policy and governance.

[13] Manuel T (2007). Economic policy and South Africa’s growth strategy.

[14] Kula N, Fryatt R (2014). Public–private interactions on health in South Africa: opportunities for scaling up. Health Policy Plan.

[15] Thadani KB (2014). Public private partnership in the health sector: boon or bane. Procedia Soc Behav Sci.

[16] Loxley J (2013). Are public–private partnerships (PPPs) the answer to Africa’s infrastructure needs?. Rev Afr Polit Econ.

[17] Walwyn DR, Nkolele AT (2018). An evaluation of South Africa’s public–private partnership for the localisation of vaccine research, manufacture and distribution. Health Res Policy Syst.

[18] Mabunda A, S, London L, Pienaar D. (2018). An evaluation of the role of an intermediate care facility in the continuum of care in Western Cape, South Africa. Int J Health Policy Manag.

[19] Lang AM (2016). Healthcare infrastructure public-private partnerships in developing countries: the Queen’Mamohato Hospital in Lesotho.

[20] Asasira J, Ahimbisibwe FJ (2018). Public-private partnership in health care and its impact on health outcomes: evidence from Ruharo Mission Hospital in Uganda. Int J Soc Sci Stud.

[21] Whyle EB, Olivier J (2016). Models of public–private engagement for health services delivery and financing in Southern Africa: a systematic review. Health Policy Plan.

[22] Suchman L, Hart E, Montagu D (2018). Public–private partnerships in practice: collaborating to improve health finance policy in Ghana and Kenya. Health Policy Plan.

[23] Moher D, Shamseer L, Clarke M, Ghersi D, Liberati A, Petticrew M (2015). Preferred reporting items for systematic review and meta-analysis protocols (PRISMA-P) 2015 statement. Syst Rev.

[24] Arksey H, O'Malley L (2005). Scoping studies: towards a methodological framework. Int J Soc Res Methodol.

[25] Márton SM, Polk G, Fiala DRC (2013). Convention on the rights of persons with disabilities.

[26] Gimigliano F, Negrini S (2017). The World Health Organization “rehabilitation 2030: a call for action”. Eur J Phys Rehabil Med.

[27] Kahonde C, Mlenzana N, Rhoda A (2010). Persons with physical disabilities’ experiences of rehabilitation services at Community Health Centres in Cape Town. S Afr J Physiother.

